# Supplementary figures and images for: Plakoglobin transmits tension across VE-cadherin for vascular leak formation and leukocyte diapedesis
Source: EMBO J. 2026 Mar 11;45(7):2210–38. doi: 10.1038/s44318-026-00732-0 (PMC13043941; doi:10.1038/s44318-026-00732-0)

**Figure 1A.**

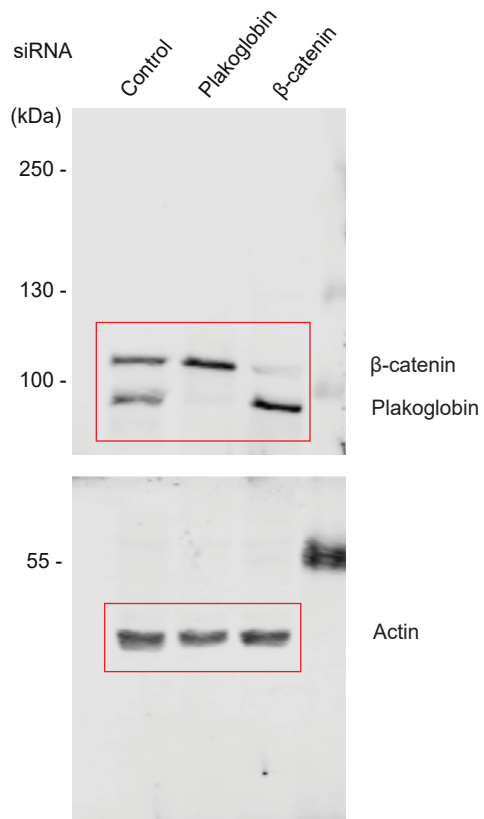

Blot was cut in half at 70 kDa

Supplement: Supplementary file 2 — Source data Fig. 1 [file 44318_2026_732_MOESM2_ESM.zip › Figure 1/1A/Figure 1A Blots.pdf]

Figure 2A.

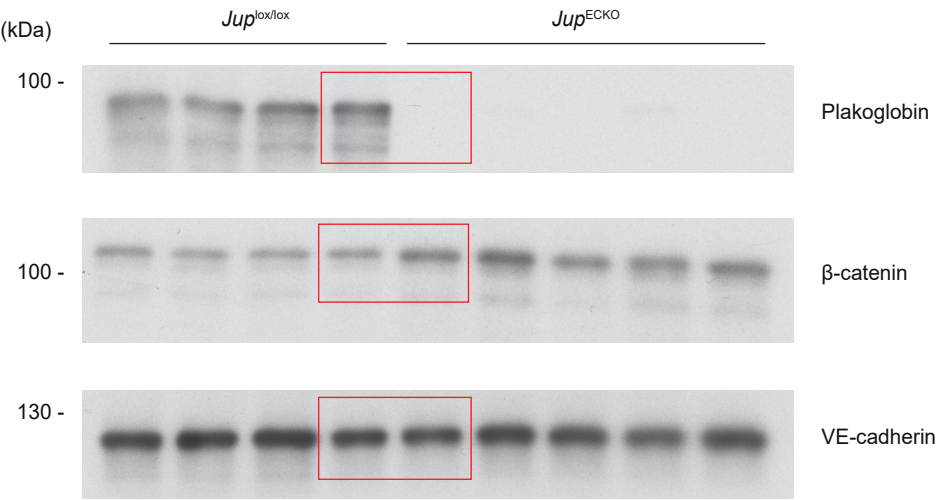

Supplement: Supplementary file 3 — Source data Fig. 2 [file 44318_2026_732_MOESM3_ESM.zip › Figure 2/2A/Figure 2A blots.pdf]

Figure 2B.

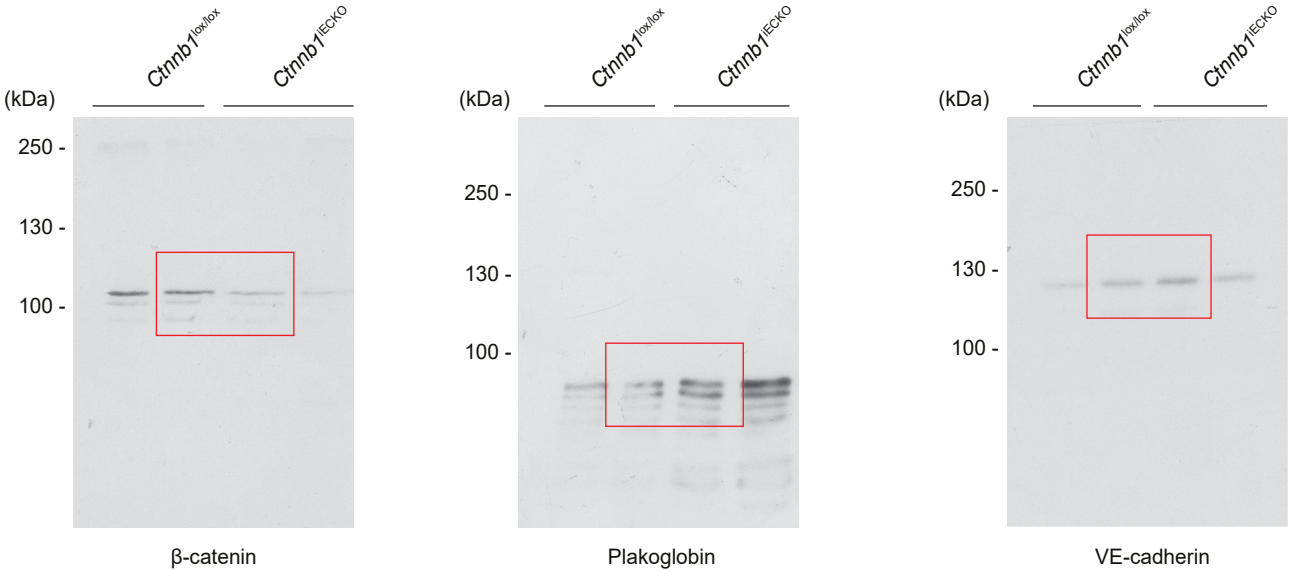

Supplement: Supplementary file 3 — Source data Fig. 2 [file 44318_2026_732_MOESM3_ESM.zip › Figure 2/2B/Figure 2B blots.pdf]

**Figure 4C.**

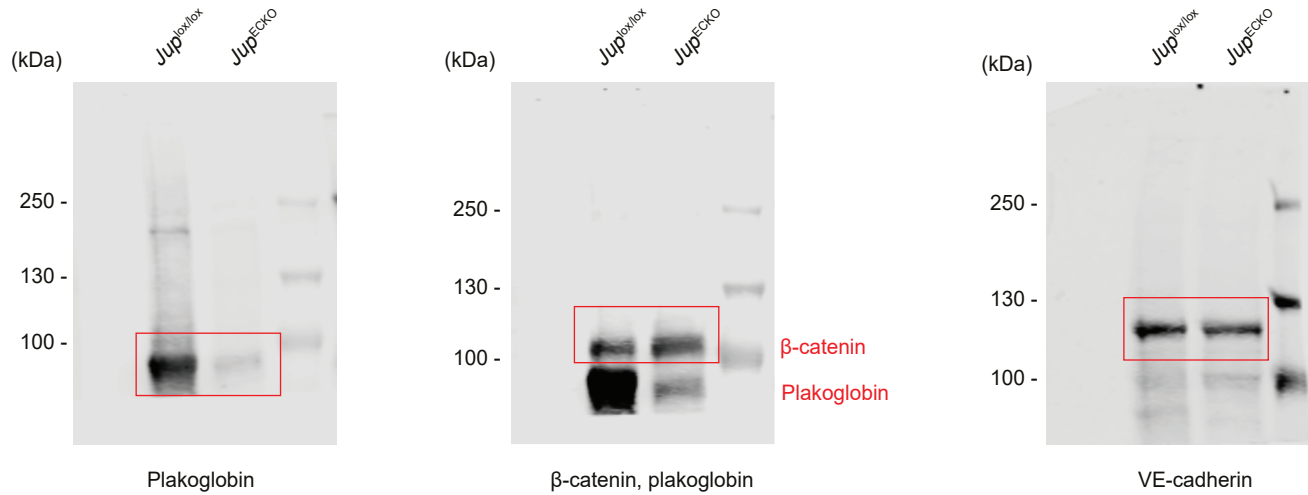

Supplement: Supplementary file 5 — Source data Fig. 4 [file 44318_2026_732_MOESM5_ESM.zip › Figure 4/4C/Figure 4C blots.pdf]

**Figure 4D.**

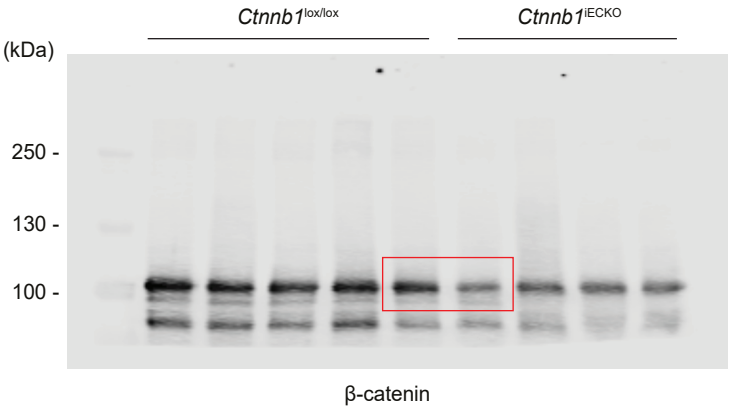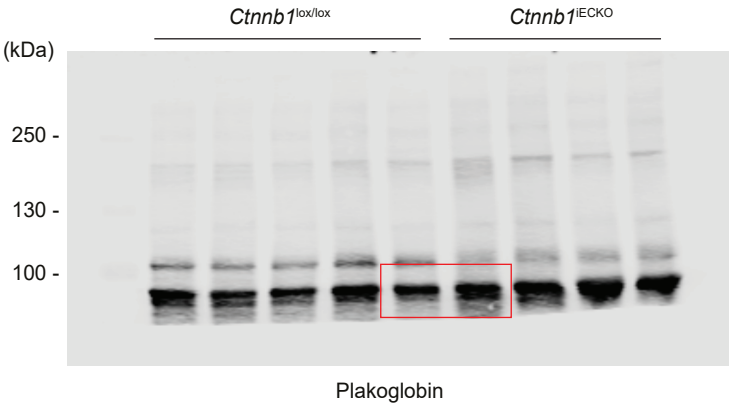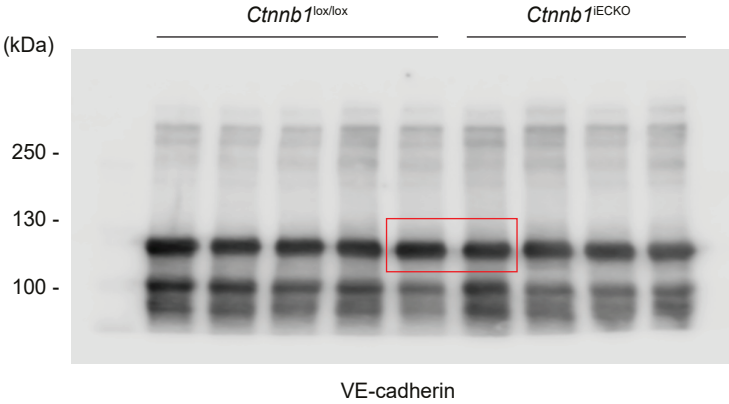

Supplement: Supplementary file 5 — Source data Fig. 4 [file 44318_2026_732_MOESM5_ESM.zip › Figure 4/4D/Figure 4D blots.pdf]

Figure 5B.

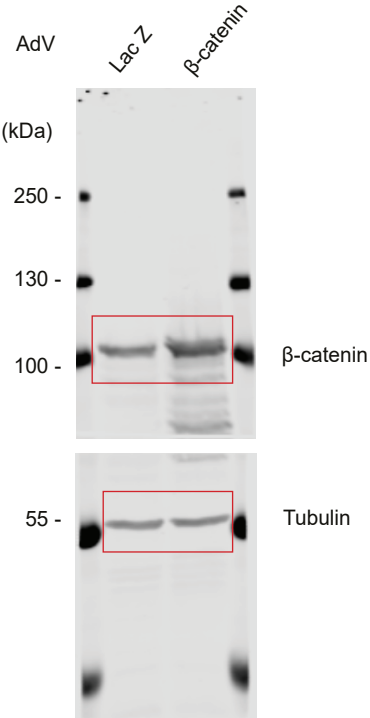

Blot was cut in half at 70 kDa

Supplement: Supplementary file 6 — Source data Fig. 5 [file 44318_2026_732_MOESM6_ESM.zip › Figure 5/5B/Figure 5B blots.pdf]

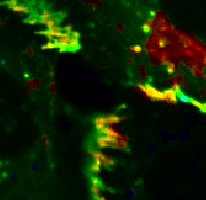

Supplement: Supplementary file 8 — Source data Fig. 7 [file 44318_2026_732_MOESM8_ESM.zip › Figure 7/7D/FLIM/si-beta-catenin.bmp]

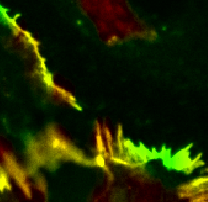

Supplement: Supplementary file 8 — Source data Fig. 7 [file 44318_2026_732_MOESM8_ESM.zip › Figure 7/7D/FLIM/si-Control.bmp]

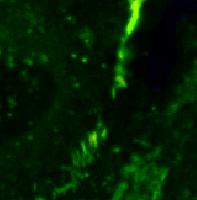

Supplement: Supplementary file 8 — Source data Fig. 7 [file 44318_2026_732_MOESM8_ESM.zip › Figure 7/7D/FLIM/si-Plakoglobin.bmp]

Figure 7E.

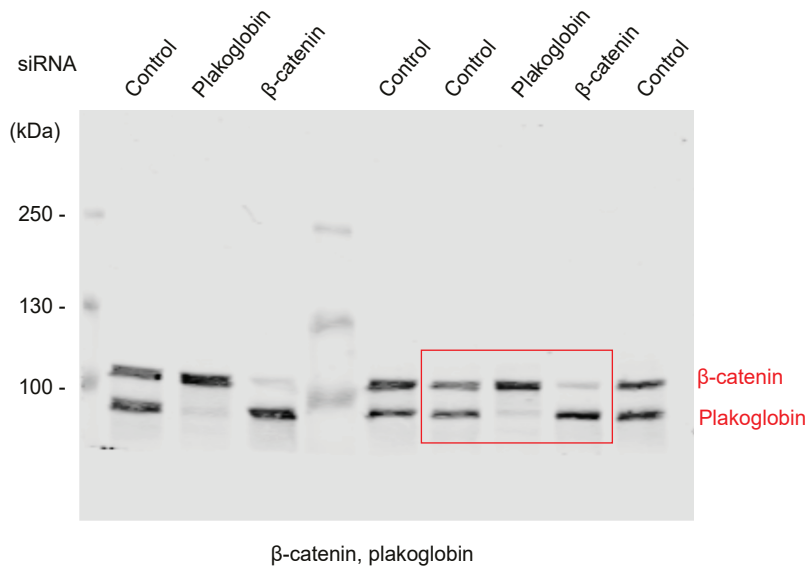

Blot was cut in half at 70 kDa

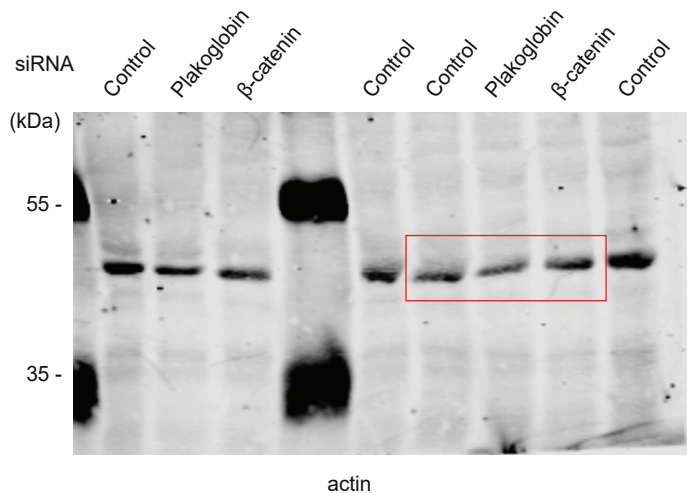

Supplement: Supplementary file 8 — Source data Fig. 7 [file 44318_2026_732_MOESM8_ESM.zip › Figure 7/7E/Figure 7E Blots.pdf]

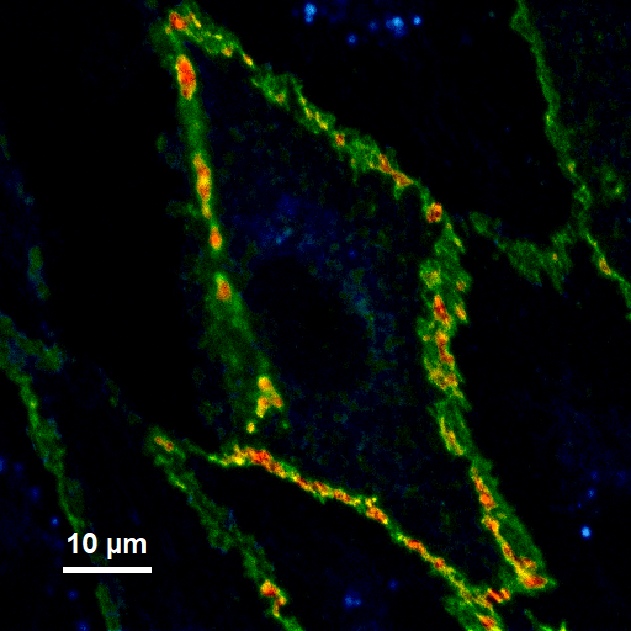

Supplement: Supplementary file 9 — Source data Fig. 8 [file 44318_2026_732_MOESM9_ESM.zip › Figure 8/8C/si-beta-catenin_histamine.bmp]

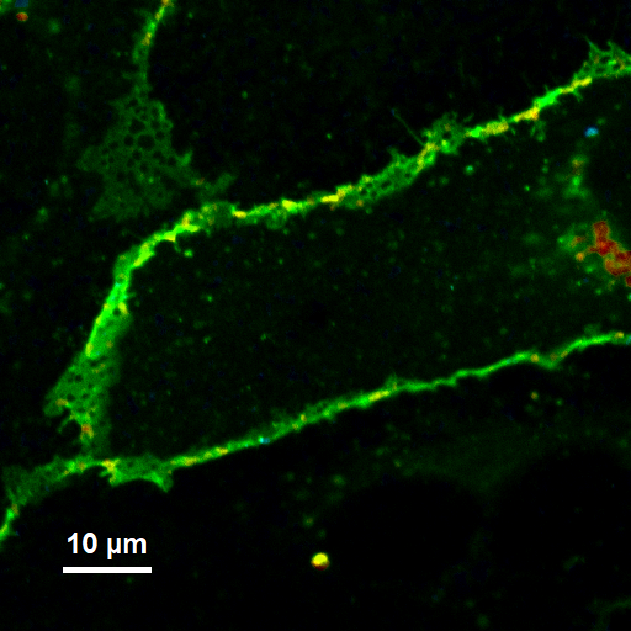

Supplement: Supplementary file 9 — Source data Fig. 8 [file 44318_2026_732_MOESM9_ESM.zip › Figure 8/8C/si-beta-catenin_untreated.bmp]

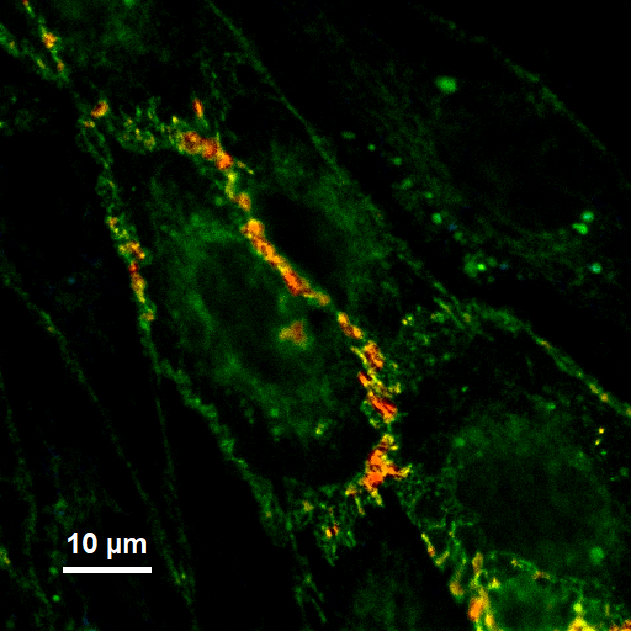

Supplement: Supplementary file 9 — Source data Fig. 8 [file 44318_2026_732_MOESM9_ESM.zip › Figure 8/8C/si-control_histamine.bmp]

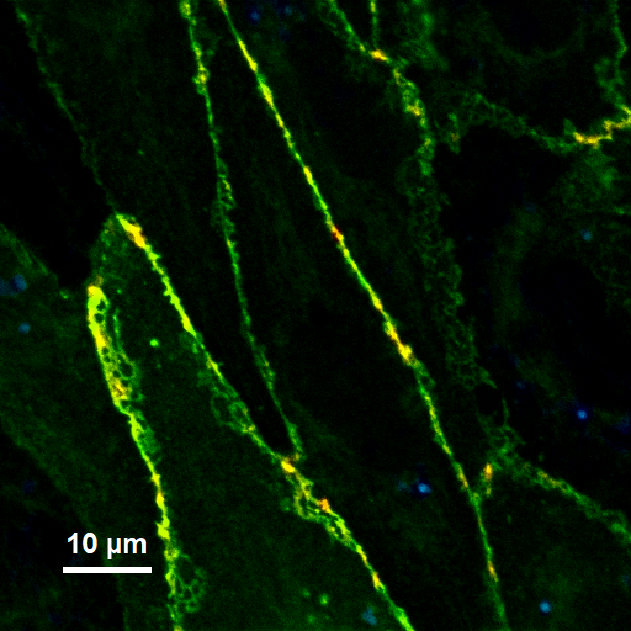

Supplement: Supplementary file 9 — Source data Fig. 8 [file 44318_2026_732_MOESM9_ESM.zip › Figure 8/8C/si-control_untreated.bmp]

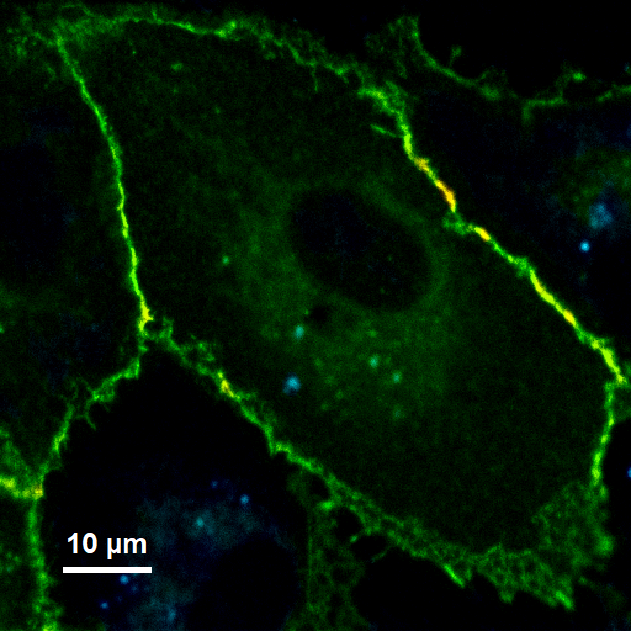

Supplement: Supplementary file 9 — Source data Fig. 8 [file 44318_2026_732_MOESM9_ESM.zip › Figure 8/8C/si-plakoglobin_histamine.bmp]

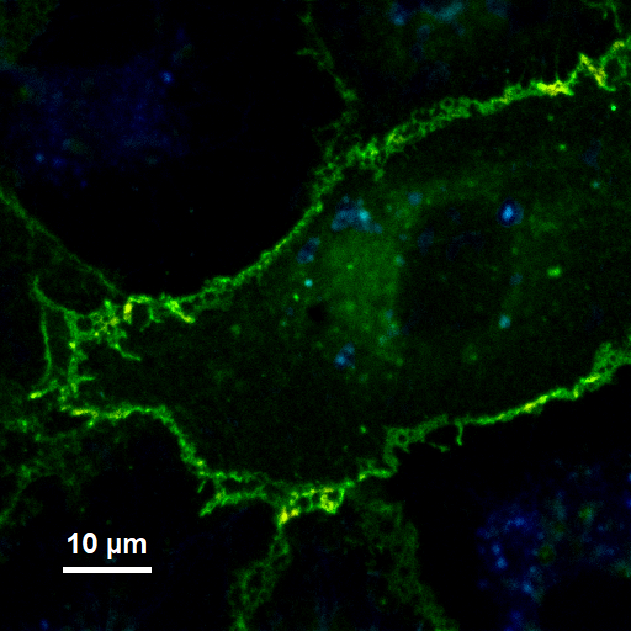

Supplement: Supplementary file 9 — Source data Fig. 8 [file 44318_2026_732_MOESM9_ESM.zip › Figure 8/8C/si-plakoglobin_untreated.bmp]

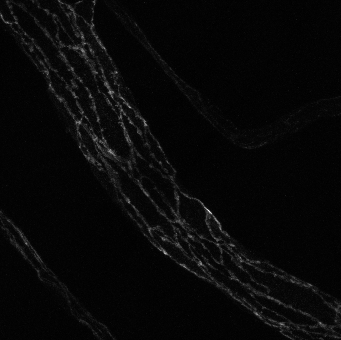

Supplement: Supplementary file 10 — Source data Fig. 9 [file 44318_2026_732_MOESM10_ESM.zip › Figure 9/9B/C57BL6_PECAM1.tif]

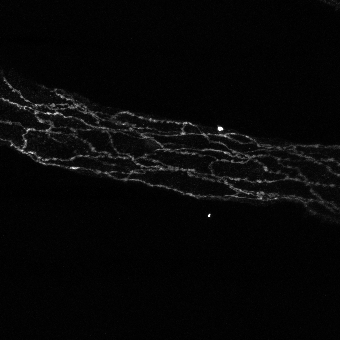

Supplement: Supplementary file 10 — Source data Fig. 9 [file 44318_2026_732_MOESM10_ESM.zip › Figure 9/9B/VEC-TS_PECAM1.tif]

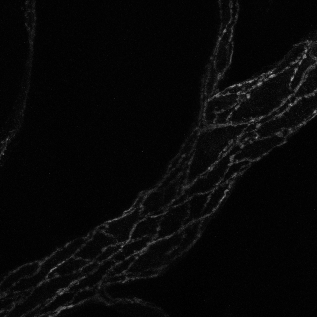

Supplement: Supplementary file 10 — Source data Fig. 9 [file 44318_2026_732_MOESM10_ESM.zip › Figure 9/9B/VEC-TS-NF_PECAM1.tif]

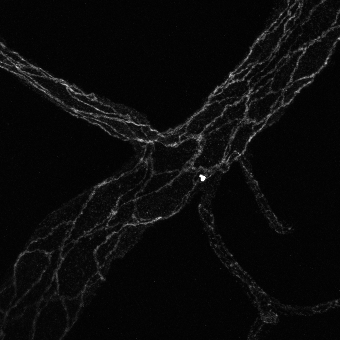

Supplement: Supplementary file 10 — Source data Fig. 9 [file 44318_2026_732_MOESM10_ESM.zip › Figure 9/9B/VEC-TS-YPet_PECAM1.tif]

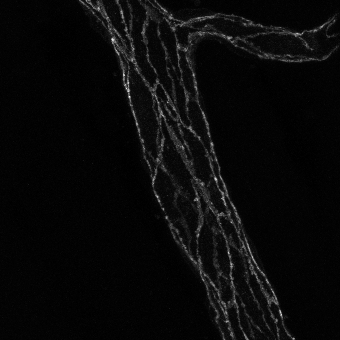

Supplement: Supplementary file 10 — Source data Fig. 9 [file 44318_2026_732_MOESM10_ESM.zip › Figure 9/9B/VEC-WT_PECAM1.tif]

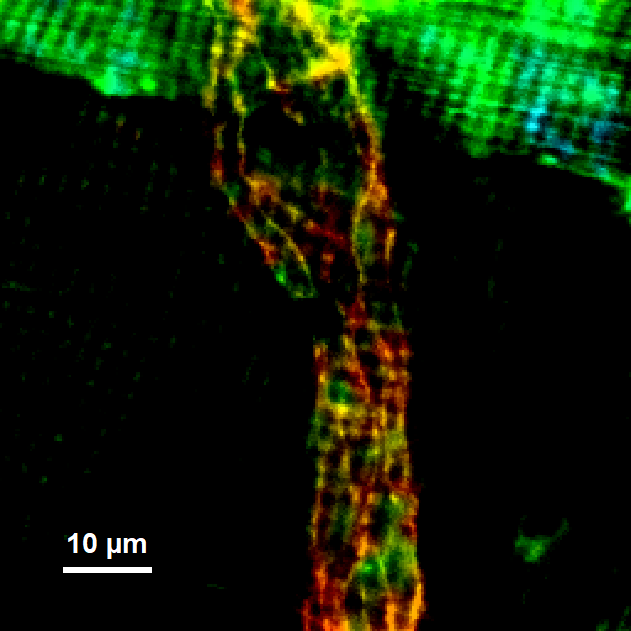

Supplement: Supplementary file 11 — Source data Fig. 10 [file 44318_2026_732_MOESM11_ESM.zip › Figure 10/10C/VEC-TS_Histamine.bmp]

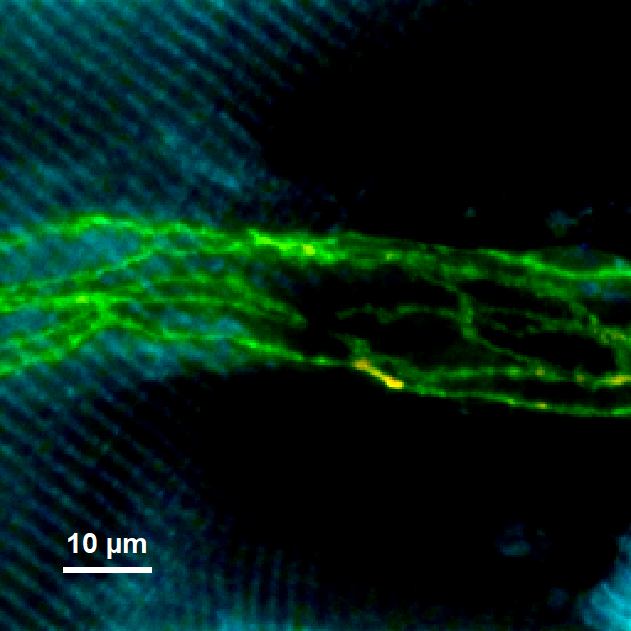

Supplement: Supplementary file 11 — Source data Fig. 10 [file 44318_2026_732_MOESM11_ESM.zip › Figure 10/10C/VEC-TS_PBS.bmp]

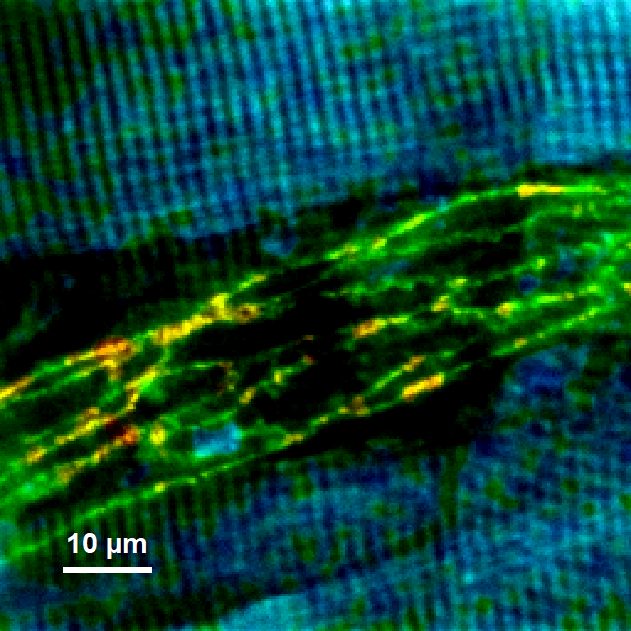

Supplement: Supplementary file 11 — Source data Fig. 10 [file 44318_2026_732_MOESM11_ESM.zip › Figure 10/10C/VEC-TS-NF_Histamine.bmp]

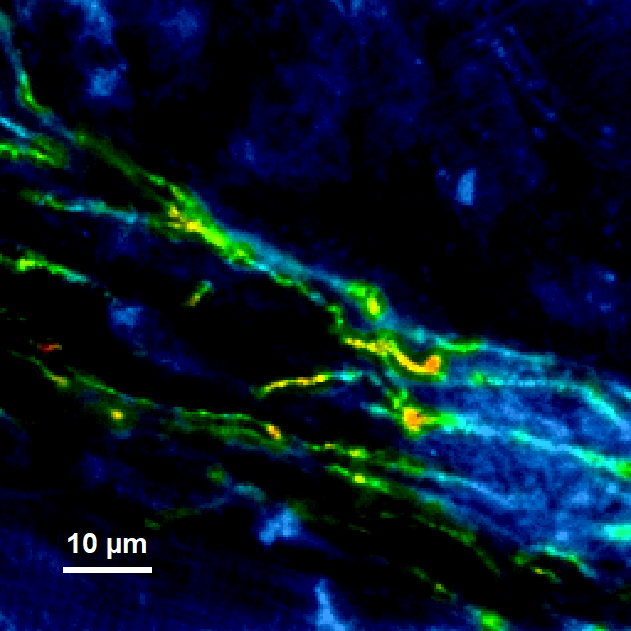

Supplement: Supplementary file 11 — Source data Fig. 10 [file 44318_2026_732_MOESM11_ESM.zip › Figure 10/10C/VEC-TS-NF_PBS.bmp]

Figure 11A.

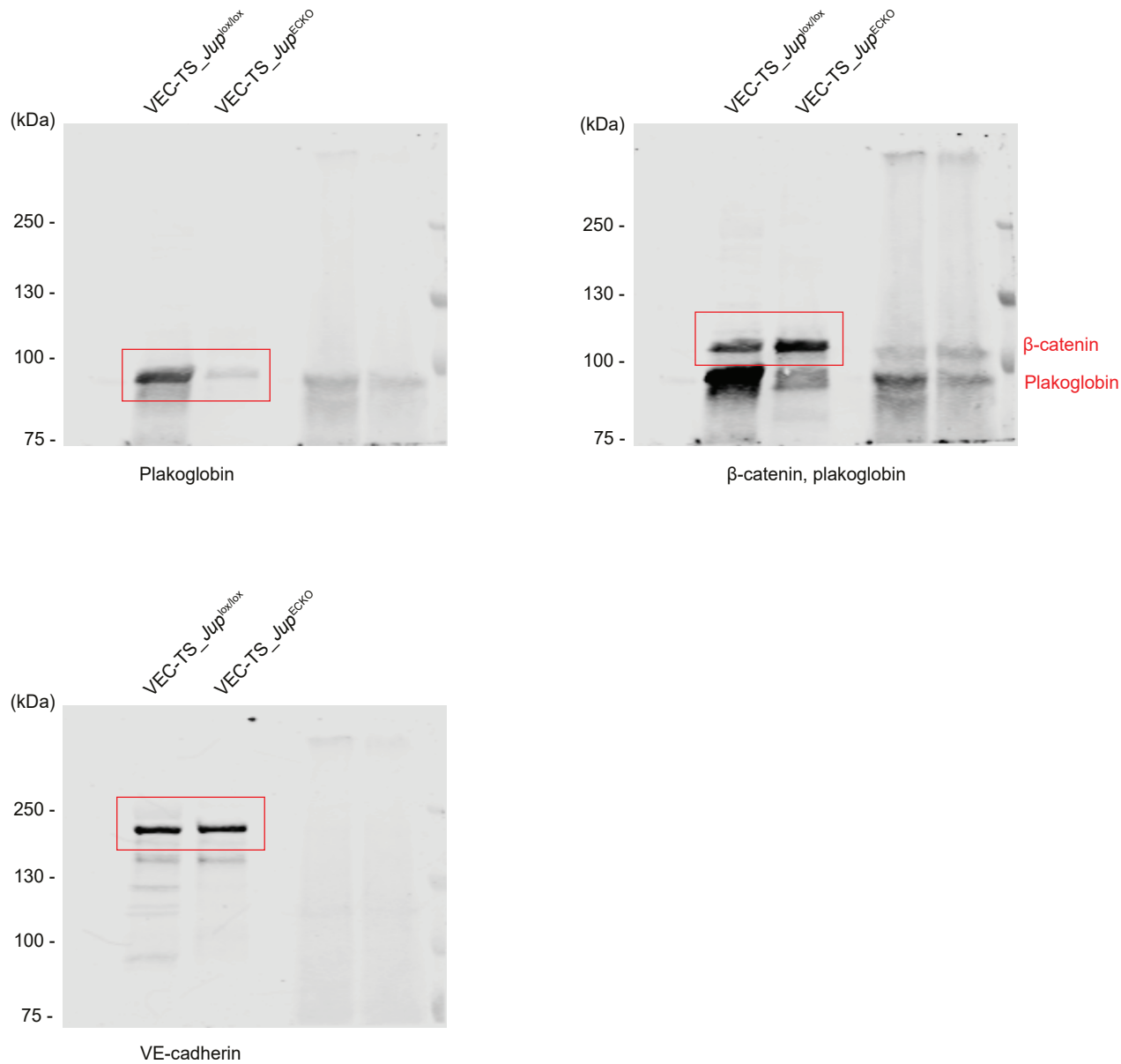

Supplement: Supplementary file 12 — Source data Fig. 11 [file 44318_2026_732_MOESM12_ESM.zip › Figure 11/11A/Figure 11A blots.pdf]
