# Supplementary material for: Plakoglobin transmits tension across VE-cadherin for vascular leak formation and leukocyte diapedesis
Source: EMBO J. 2026 Mar 11;45(7):2210–38. doi: 10.1038/s44318-026-00732-0 (PMC13043941; doi:10.1038/s44318-026-00732-0)
Supplement: Supplementary file 13 — Expanded View Figures [file 44318_2026_732_MOESM13_ESM.pdf]

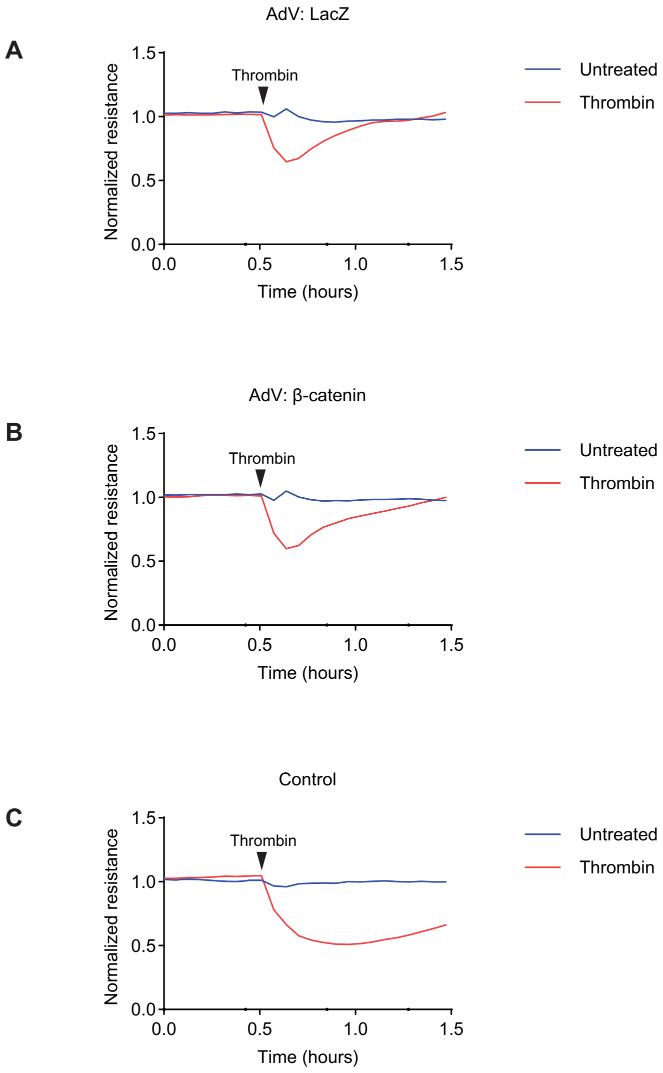

Figure EV2. Electrical resistance measurements of HUVEC exposed to adenovirus vectors.

(**A–C**) HUVEC were transduced with LacZ (**A**), or β-catenin (**B**) adenovirus, or left untransduced as control (**C**), and grown to confluency on fibronectin-coated electrode arrays. Monolayers were subsequently stimulated with 1 U/mL thrombin (red lines) or left untreated (blue lines), and transendothelial electrical resistance was recorded over time by ECIS. Representative graphs show resistance after normalization to the resistance of HUVEC monolayers before thrombin treatment.

© The Author(s)

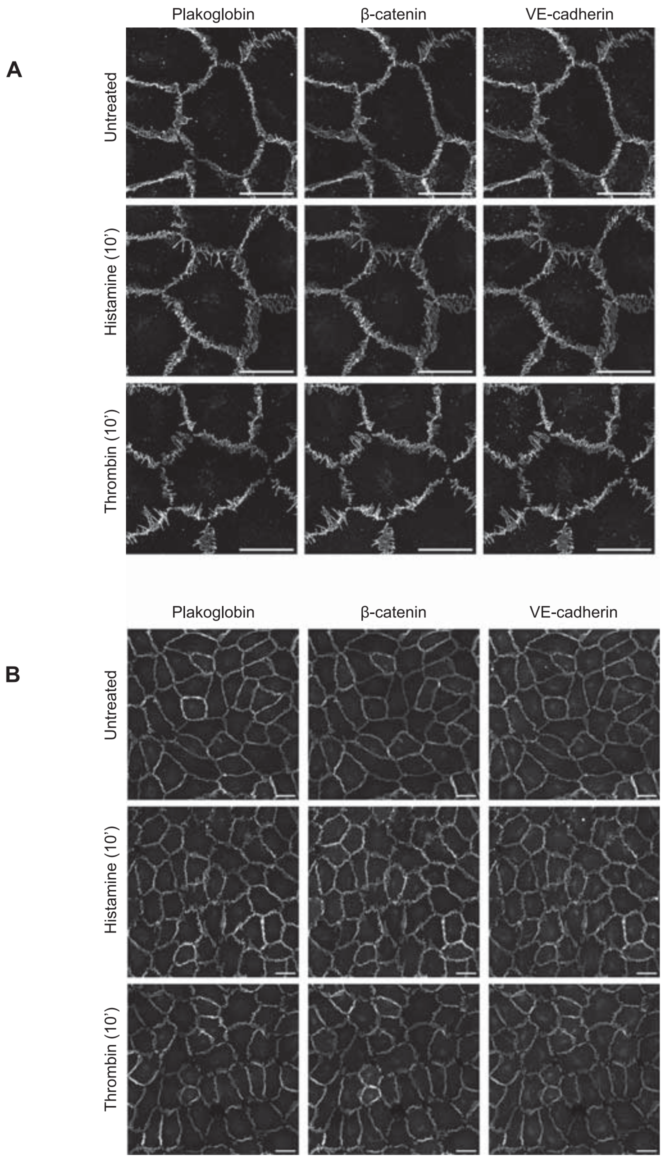

Figure EV3. Plakoglobin or β-catenin distribution showed no major differences within inflamed and control endothelial monolayers.

(**A**, **B**) HUVEC were treated with histamine (100 µM, Sigma-Aldrich) or thrombin (1 U/ml, CalBiochem) for 10 min, or left untreated with equivalent volumes of vehicle (media) as controls. Cells were subsequently fixed, permeabilized and stained for plakoglobin, β-catenin, and VE-cadherin, all visualized in grayscale to ensure unbiased comparative analysis. Higher magnification images (63X) were acquired using a Zeiss LSM 980 confocal microscope equipped with an Airyscan detector (**A**), while lower magnification images (40X) were captured on a Zeiss LSM 880 confocal microscope (**B**). Scale bars, 25 µm.

 © The Author(s)

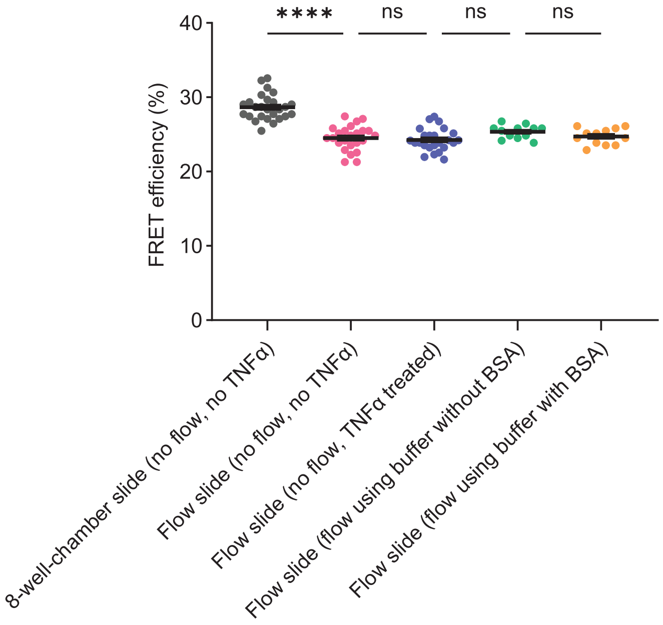

Figure EV4. Variability in FRET efficiency (%) of VEC-TS-NF construct.

FRET efficiency at endothelial junctions was quantified in HUVEC expressing VEC-TS-NF and cultured in ibidi 8-well chambers or flow chamber slides under distinct conditions, including TNFα stimulation (4 h) and exposure to flow (1 dyn/cm²) using flow buffer with or without BSA. Cells were fixed with 4% PFA, washed with PBS, and analyzed by FLIM. Data information: Graph shows mean ± SEM from ($n = 26, 24, 24, 12, 12$) measurements. ****$P < 0.0001$, one-way ANOVA. ns, not significant.
